# ANTHROPOMETRIC AND DIETARY ASSESSMENT OF PATIENTS WITH GLYCOGENOSIS TYPE I

**DOI:** 10.1590/1984-0462/2021/39/2020046

**Published:** 2021-02-05

**Authors:** Natália Bauab Jorge, Adriana Maria Alves de Tommaso, Gabriel Hessel

**Affiliations:** a Universidade Estadual de Campinas, Campinas, SP, Brazil.

**Keywords:** Glycogen storage disease type I, Anthropometry, Diet, Doença de depósito de glicogênio tipo I, Antropometria, Dieta

## Abstract

**Objective:**

To perform anthropometric and dietary evaluation of patients with glycogenosis type Ia and Ib.

**Methods:**

This cross-sectional study is composed of a sample of 11 patients with glycogenosis divided into two subgroups according to the classification of glycogenosis (type Ia=5 and type Ib=6), aged between 4 and 20 years. The analyzed anthropometric variables were weight, height, body mass index, and measures of lean and fat body mass, which were compared with reference values. For dietary assessment, a food frequency questionnaire was used to calculate energy and macronutrients intake as well as the amount of raw cornstarch consumed. Mann-Whitney U test and Fisher’s exact test were performed, considering a significance level of 5%.

**Results:**

Patients ingested raw cornstarch in the amount of 0.49 to 1.34 g/kg/dose at a frequency of six times a day, which is lower than recommended (1.75-2.50 g/kg/dose, four times a day). The amount of energy intake was, on average, 50% higher than energy requirements; however, carbohydrate intake was below the adequacy percentage in 5/11 patients. Short stature was found in 4/10 patients; obesity, in 3/11; and muscle mass deficit, in 7/11. There were no statistical differences between the subgroups.

**Conclusions:**

In patients with glycogenosis type I, there was deficit in growth and muscle mass, but no differences were found between the subgroups (Ia and Ib). Although the diet did not exceed the adequacy of carbohydrates, about 1/3 of the patients presented obesity, probably due to higher energy intake.

## RESUMO

**Objetivo:**

Realizar avaliação antropométrica e dietética de pacientes com glicogenose tipos Ia e Ib.

**Métodos:**

Estudo transversal composto de uma amostra de 11 pacientes com glicogenose divididos em dois subgrupos de acordo com a classificação da glicogenose (tipo Ia=5; tipo Ib=6), com idades entre 4 e 20 anos. As variáveis antropométricas analisadas foram peso, estatura, índice de massa corporal e medidas de massa magra e gorda, que foram comparadas com valores de referência. Para avaliação dietética, foi utilizado um questionário de frequência alimentar para cálculo de ingestão de energia e macronutrientes, além da quantidade de amido cru ingerida. Realizaram-se testes U de Mann-Whitney e exato de Fisher, com nível de significância de 5%.

**Resultados:**

Os pacientes ingeriram amido cru na quantidade de 0,49 a 1,34 g/kg/dose na frequência de seis vezes ao dia, inferior à dosagem preconizada (1,75-2,50 g/kg/dose quatro vezes ao dia). A quantidade de energia consumida foi, em média, 50% a mais que as necessidades, contudo o consumo de carboidratos foi abaixo da porcentagem de adequação em 5/11 pacientes. Baixa estatura ocorreu em 4/10 pacientes, obesidade em 3/11 e déficit de massa muscular em 7/11. Não houve diferença estatística entre os subgrupos.

**Conclusões:**

Em pacientes com glicogenose tipo I, houve déficit de crescimento e de massa muscular, mas não diferença significante entre os subgrupos (Ia e Ib). Embora a dieta não tenha ultrapassado a adequação de carboidratos, 1/3 dos pacientes apresentou obesidade, provavelmente pela maior ingestão de energia.

## INTRODUCTION

Glycogenosis type I (GSD I) is an inborn error of glycogen metabolism and is related to a deficiency in the activity of the enzyme glucose-6-phosphatase.^1^ ^,^ ^2^ It can be subdivided into two main types: type Ia, in which inactivity of the glucose-6-phosphatase enzyme is observed; and type Ib, in which there is a defect in glucose-6-phosphate transport.^1^ ^,^ ^3^ Its incidence is estimated at 1: 100,000 live births.^4^

As a consequence, GSD I can lead to the accumulation of glycogen in the liver, kidneys, and intestinal mucosa, in addition to metabolic consequences such as hypoglycemia (which can lead to seizures), lactic acidosis, hyperlipidemia, and hyperuricemia. Moreover, clinical signs, such as doll-like facies and hepatomegaly, are frequent. In the long term, kidney complications, pulmonary hypertension, and even hepatic adenomas may appear. Glycogenosis (GSD) type Ib differs from type Ia by the recurrent infections associated with neutropenia and neutrophil dysfunction presented by the patients.^2^ ^,^ ^3^ ^,^ ^5^ ^,^ ^6^ ^,^ ^7^

Diet therapy is paramount and aims to prevent hypoglycemia, improve metabolic control, ensure proper growth, and postpone long-term complications.^1^ ^,^ ^2^ ^,^ ^5^ ^,^ ^6^ It consists in providing a fractional nutrition throughout the day, with slow-absorbing carbohydrates (such as raw cornstarch) at intervals for maintaining blood glucose, in addition to restricting lactose, sucrose, and fructose in the diet . Considering that this diet is limited, it is often necessary to consume vitamin and mineral supplements.^1^ ^,^ ^2^ ^,^ ^7^ ^,^ ^8^

As for the nutritional profile, GSD I patients may present growth deficit and, in some cases, short stature.^1^ Excess weight gain may be due to the excessive supply of energy and/or cornstarch.^5^ ^,^ ^6^

The follow-up of nutritional status can be carried out by monitoring the measurements of weight, height, and body mass index, in addition to mid upper arm circumference and skinfolds, which are useful in assessing lean and fat mass.^5^ ^,^ ^9^

Considering the rarity of the disease and its metabolic consequences, nutritional treatment is paramount for these patients. Thus, the objective of this research was to perform the anthropometric and dietary evaluation of patients with glycogenosis types Ia and Ib followed up in an outpatient basis at a tertiary healthcare service.

## METHOD

This is a descriptive and cross-sectional study carried out on patients with GSD I followed up at the clinic of pediatric hepatology and inborn errors of metabolism from a tertiary hospital. Of the total of 18 patients with GSD I who were followed up, seven were excluded (two for not having a confirmed diagnosis; one for not having completed the exams; one for choosing not to participate in the research; and three for not responding to the call). The diagnosis of the disease of these patients was established based on clinical history, biochemical tests, liver biopsy analyzed by optical and electronic microscopy, and molecular study.

Patients were divided into two groups according to the classification of glycogenosis, based on the clinical condition and on the study of gene mutation by molecular analysis (type Ia=5 patients; type Ib=6 patients). In the clinical history and biochemical tests, hepatomegaly, hypoglycemia (sometimes with secondary seizure), hypercholesterolemia, hypertriglyceridemia, hyperuricemia, and increased lactate levels were observed. Some patients had doll-like facies. Patients with type Ib also had neutropenia and recurrent infections. Liver biopsy demonstrated hepatocytes with clear cytoplasm, steatosis, and thickened cytoplasmic membrane resembling a plant cell due to the displacement of organelles towards the cell periphery caused by excessive glycogen accumulation. In the electron microscopy, an abundant deposition of cytoplasmic and nuclear glycogen was observed. In the molecular analysis performed with eight patients, five of them showed mutations compatible with group Ia in exons of the G6PC gene, and three mutations compatible with group Ib in exons of the SLC37A4 gene.

All patients consumed raw cornstarch as part of the treatment and had regular consultations with a nutritionist for nutritional assessment and guidance on the consumption of this food, dietary restrictions on lactose, sucrose, and fructose, especially for the younger patients,^5^ ^,^ ^10^ ^,^ ^11^ in addition to dietary adjustments and specific guidelines when necessary.

Patients were invited to participate in the research after their routine consultations at the healthcare service. At that moment, anthropometric and dietary data were collected by the main researcher. The variables collected for anthropometric analysis, following the techniques described by Lohman et al.,^12^ were: weight, height, mid upper arm circumference (MUAC) and triceps skinfold thickness (TST) - for the latter, the Lange^®^ adipometer (Beta Technology, Santa Cruz, CA, United States of America) was used. Based on these data, the following variables were calculated: body mass index (BMI=weight/height^2^); arm muscle circumference (AMC); bone-free muscle area (UMAc), and upper arm fat area (UFA), by using the Frisancho’s formulas.^13^ These measures were compared with reference values in percentiles. For patients aged up to 20 years (10/11 patients), the Z scores for height according to age (height-for-age) and body mass index according to age (BMI-for-age) were also calculated. For patients aged up to 10 years, the Z score for weight according to age (weight-for-age) was also calculated. The WHO AntroPlus v. 1.0.4 software (World Health Organization, Geneva, Switzerland) was used for estimating the Z scores.

For dietary assessment, an interview was conducted with patients themselves, in the case of adolescents and adults, or with the guardians/parents, in the case of children, using a semi-quantitative food frequency questionnaire (FFQ) adapted from Ribeiro et al.^14^ The questionnaire contained a list with different categories of food, intake frequency, and consumed portion in order to assess the patient’s usual intake based on the previous 30 days. To calculate the diet based on the questionnaire, the Dietpro 5i software (Dietpro, Viçosa, MG, Brazil) was used, which allowed for the determination of the amounts of energy, carbohydrates, proteins, and fats ingested. Then, the percentage of adequacy of these macronutrients was calculated, and the values were compared with those established by the *Protocolo Brasileiro de Dietas: Erros Inatos de Metabolismo* [Brazilian Diet Protocol: Inborn Errors of Metabolism].^10^ According to this protocol, the recommended value for carbohydrates must be between 60-65% of the total energy value; for proteins, between 10-15%; and for fats, between 20-25%. Energy requirements were calculated using the Estimated Energy Requirements (EER) formulas, established by the Dietary References Intakes (DRIs).^15^

All data were collected and compiled by the researchers, and the statistical analysis was performed using the *Statistical Package for the Social Sciences* (SPSS) program (version 20.0). Frequency and descriptive statistics were performed. In addition, for continuous variables, the Mann-Whitney U test was employed; for categorical variables, the Fisher’s exact test was used, both with a 5% significance level.

The research was approved by the Research Ethics Committee of the School of Medical Sciences - Universidade Estadual de Campinas (Unicamp), Certificate of Presentation for Ethical Consideration (CAAE) No. 11737312.1.0000.5404.

## RESULTS

The sample of this study was composed of 11 patients with GSD I, accounting for six female patients and five male patients. The age ranged from 4 years and 6 months to 20 years and 6 months, and the follow-up time, from 3 years and 5 months to 17 years and 2 months. Data for dietary assessment of each patient are shown in Table 1.

**Table 1:** Characteristics of the dietary assessment of each patient with glycogenosis type I.

Patient	Age (years and months)	Sex	Type of disease	Energy intake *vs.* EER*	Contribution of macronutrients ingested				Cornstarch (g/kg/dose)
					CHO (%)	Protein (%)	Lipídeo (%)	Cornstarch (%)**	
1	5y10m	F	Ib	150.1	59.6	17.0	23.4	23.2	1.0
2	5y2m	M	Ia	115.4	62.0	12.9	24.8	30.9	1.0
3	7y1m	M	Ib	137.9	52.2	19.7	28.1	24.3	0.9
4	8y	F	Ia	148.2	65.2	18.1	16.7	29.3	1.0
5	17y10m	M	Ib	89.5	64.0	15.6	20.4	40.6	0.5
6	13y5m	M	Ib	208.7	64.3	13.9	21.8	32.1	0.9
7	12y10m	F	Ia	154.7	67.5	13.8	18.7	35.7	0.8
8	20y6m	M	Ib	103.4	52.5	26.4	21.2	22.1	0.7
9	8y9m	F	Ib	224.7	56.0	19.6	24.4	25.2	1.3
10	17y2m	F	Ia	150.5	63.0	15.3	21.7	31.9	0.7
11	4y6m	F	Ia	219.3	55.3	20.6	24.2	21.6	1.3

F: female; M: male; EER: Estimated Energy Requirements; CHO: carbohydrates; *percentage calculated by comparing the estimation of energy intake with EER; **the percentage of cornstarch refers to the cornstarch contribution to the total energy of the diet.

Patients were followed up by a nutritionist from the healthcare service who guided the diet therapy; however, during the research, only the oldest patient lost to follow-up. All patients orally consumed raw cornstarch diluted in water as part of the treatment, with a median intake of six times/day (with a minimum of four and a maximum of eight times/day), at regular intervals. Regarding the frequency of meals and snacks throughout the day, patients had a median of six meals/day.

The results of the dietary assessment by the FFQ are presented in Table 2. As for the calories ingested, when compared with reference values, both groups consumed an average of 50% more than their estimated requirements. When analyzing the contribution of each macronutrient to the diet, the proportion of carbohydrates for type Ia complied with the reference values; for patients with type Ib, this value was below the recommendation. The distribution of proteins in the diet was higher than the recommended value in both groups of patients, whereas fat intake was in line with the range of recommended values. Nevertheless, there was no statistically significant difference between both groups.

**Table 2:** Mean and median of the amounts of energy and nutrients ingested by patients with glycogenosis type I, according to the food frequency questionnaire.

	Glycogenosis I n=11	Glycogenosis Ia n=5	Glycogenosis Ib n=6	p-value
	Mean (median)			
Age (months)	132.1 (105.0)	114.4 (96.0)	146.8 (133.0)	0.27
Energy				
Intake (kcal)	2,371.0 (2,425.3)	2,173.8 (2,335.5)	2,535.3 (2,606.3)	0.27
Intake *vs.* EER (%)*	154.7 (150.0)	157.6 (150.5)	152.3 (144.0)	0.58
Intake (kcal/kg weight)	79.3 (71.6)	79.8 (61.4)	78.9 (83.4)	0.86
Macronutrients				
Carbohydrate (%)	60.1 (62.0)	62.6 (63.0)	59.0 (57.8)	0.20
Protein (%)	17.5 (17.0)	16.1 (15.3)	18.7 (18.3)	0.27
Fat (%)	22.3 (21.8)	21.2 (21.7)	23.2 (22.6)	0.47
Raw cornstarch				
% energy from raw cornstarch	28.8 (29.3)	29.9 (30.9)	27.9 (24.7)	0.33

EER: Estimated Energy Requirements; *percentage calculated by comparing the estimation of energy intake with the EER.

The patients’ intake of macronutrients was also evaluated, comparing it with reference values based on the dietary assessment. When employing the Fisher’s exact test, no significant difference was observed between GSD Ia and Ib. However, despite the patients’ intake of cornstarch being part of the treatment, only 2/11 patients accounted for carbohydrates above the reference values. The value of protein intake in 8/11 patients was higher than the reference value, and five of these patients were of the subtype Ib. As for fat, 8/11 patients had intake according to the reference value.

Concerning the results of the anthropometric evaluation, 5/6 patients had adequate weight-for-age as evaluated by the Z score, whereas for BMI-for-age, 8/11 were eutrophic and 3/11 were overweight (2/11 being of type Ia). As for height, 4/10 patients presented low height-for-age - two patients who presented very low height-for-age had glycogenosis type Ib. Only one patient (1/10) had short stature associated with obesity. However, when comparing both groups, there was no statistical difference between them, as demonstrated in Table 3. No association was found between anthropometric parameters and deficiency or excess of any macronutrient, according to the Fisher’s exact test.

**Table 3:** Values of means and medians of the anthropometric assessment of patients with glycogenosis.

	Glycogenosis I	Glycogenosis Ia	Glycogenosis Ib	p-value
	n=11	n=5	n=6	
	Mean (median)			
Z score BMI-for-age n=10*	0.97 (0.74)	1.10 (0.99)	0.85 (0.61)	0.75
Z score weight-for-age n=6**	-0.39 (-0.59)	0.41 (0.32)	-1.19 (-0.62)	0.27
Z score height-for-age n=10*	-1.79 (-1.46)	-1.25 (-1.11)	-2.35 (-1.70)	0.25
MUAC (cm)	22.3 (21.4)	19.9 (19.7)	24.4 (23.9)	0.52
AMC (cm)	18.9 (16.6)	16.1 (16.6)	21.3 (20.1)	0.46
UMAc (cm^2^)	23.5 (15.3)	13.8 (15.3)	31.5 (23.2)	0.27
TST (mm)	10.9 (11.0)	12.2 (12.0)	9.8 (8.0)	0.23
UFA (cm^2^)	9.2 (9.0)	8.6 (9.0)	9.8 (10.1)	0.58

BMI-for-age: body mass index according to age; weight-for-age: weight according to age; height-for-age: height according to age; MUAC: mid upper arm circumference; AMC: arm muscle circumference; UMAc: bone-free muscle area; TST: triceps skinfold thickness; UFA: upper arm fat area; *not calculated for adult patient; **only calculated for patients aged up to 10 years.

**Graph 1:**
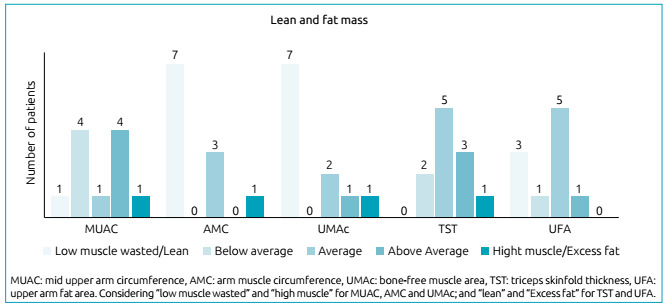
Profile of the evaluation of lean and fat mass of patients with glycogenosis type I.

The assessment of patients’ nutritional status based on lean and fat mass is shown in Graph 1.

## DISCUSSION

Although GSD I can have a strong impact on the patient’s nutritional status and diet therapy is paramount, there are few studies that address the quantitative assessment of the diet of these patients in addition to cornstarch intake. This study outstands in the literature for specifically addressing such aspect.

Nutritional therapy is the first line of treatment and aims, in the first place, to avoid fasting in order to prevent hypoglycemia by the fractional consumption of complex carbohydrates, with raw cornstarch consisting in the main carbohydrate. Although this is part of the treatment, the consumed amounts vary from patient to patient. The investigation conducted by Santos et al.^7^ shows raw cornstarch intake values of 0.5-2.5 g/kg/dose; in the present study, this value ranged from 0.49 to 1.34 g/kg/dose. When compared with the *Protocolo Brasileiro de Dietas: Erros Inatos de Metabolismo*,^10^ both the mean and the individual values of the patients in this study were below the recommended values (1.75-2.5 g/kg/dose); however, the recommended number of doses (four times/day) is lower than the number verified in this study. Considering that the prescription of the amount of raw cornstarch must be individualized, the ideal dose should be the minimum for controlling hypoglycemia, without causing adverse effects, thus preventing the excessive consumption of raw cornstarch from leading to overweight and obesity.

Although no study has elucidated the frequency of intermediate meals and snacks ingested by patients, in the present study the mean fractionation of diets was six times/day, with this frequency accounting for one additional time per day when compared with the European guideline for the management of GSD I.^11^

Several studies address the different forms of consumption of cornstarch/carbohydrates in the treatment of glycogenosis, as well as their routes and intervals of administration; however, few studies assess the patients’ diet, both in quantitative and qualitative levels. Only the study of Bhattacharya^16^ provided energy and macronutrient assessment data from a cohort of 20 patients with GSD I. Regarding energy intake, the author reported that patients had an average of 11% more than the Estimated Average Requirement. Patients in the current investigation also showed higher intake compared with energy requirements, with average values 50% above the values calculated by DRIs.

Feillet et al.^17^ analyzed the energy expenditure by indirect calorimetry of seven patients with GSD Ia and observed that the patients’ basal metabolic rate was increased by 16%, when compared with predictive values, and by 25%, in relation to the control group. According to the authors, this is due to increase in the cell masses of these patients (hepatomegaly, for instance), in addition to the fact that glucose production still occurs through alternative routes. In the present study, this increase in basal metabolic rate could explain the fact that the total energy value of the diet of 10/11 patients exceeds their requirements, and only three of them are obese.

Regarding the intake of macronutrients, the results of this study differed from those reported by Bhattacharya.^16^ The author found that 13/20 individuals had an intake higher than the recommendation for carbohydrates, and 9/11 had an intake below the recommendations for proteins and fats. In this research, most patients presented values of carbohydrate according to the reference values or below them, whereas the values of protein and fat were in accordance with the recommendations or above them. Bhattacharya^16^ suggests that the increased intake of carbohydrates, mainly from raw cornstarch, can lead to satiety and interfere with the intake of other foods. Considering that in the present study all patients were followed up by a nutritionist, the diet was fractionated and had lower doses of cornstarch. These factors may have contributed to the difference between both studies.

Although the recommendations for patients with glycogenosis only refer to the adequacy percentage of each macronutrient, they can mask the total amounts ingested by the patients. Often, the proportion of nutrients may be adequate, but the total amounts are excessive or insufficient. Furthermore, the recommendations do not guarantee the quality of the food, considering that a diet can be of poor quality and still be in accordance with the recommended percentages.

Concerning the profile of the patients’ nutritional status in the present analysis, the results differed from the study conducted by Däublin et al.,^18^ in which 23 patients with GSD I followed a restrictive diet and none of them were obese. In the research carried out by Schwahn et al.,^19^ only one out of 19 patients was obese, and patients with type Ia had lower weight compared with patients of type Ib, which was not observed in this study.

Several studies, such as that conducted by Rake,^20^ have also shown that short stature is common among glycogenosis patients. Chen^21^ also pinpointed that patients who did not undergo a treatment had a severe deficit. Likewise, according to the results of Melis et al*.* ^22^, patients with type Ib had greater deficit in height, whereas Schwahn et al*.* ^19^ identified that patients with type Ia had shorter stature.

In a Brazilian study, Santos et al.^7^ evaluated the anthropometry of patients with GSD I and observed that 16/21 patients were overweight (six of them were obese), and only 4/21 had short stature, thus associating a tendency for higher Z scores for height with higher BMI-for-age values. Conversely, the present study found a lower frequency of overweight and a higher frequency of short stature. This can be justified by the amount of raw cornstarch intake. In the study conducted by Santos et al.,^7^ this value is higher, which could have led to a lower growth deficit and to a greater weight gain, when undergoing an intensive diet therapy; however, the study did not evaluate the patients’ diet.

According to some analyses, the initiation of diet therapy and the good metabolic control of the disease enable an accelerated growth, whereas untreated patients have slow growth. In addition, both weight and height can be close to the 50^th^ percentile;^18^ ^,^ ^23^ ^,^ ^24^ nevertheless, Daeschel et al.^24^ emphasized that, when considering weight-for- height, patients were overweight. In a case study, Karnsakul et al.^25^ managed to revert short stature in a patient with GSD Ia, who even reached his target height by adequate metabolic control and diet therapy, but this resulted in obesity.

A possible explanation for the greater deficit in height in patients with type Ib, as verified in this study, may be related to the fact that they have neutropenia and recurrent infections. Consequently, they are more exposed to hospitalizations, changes in dietary patterns, and use of medications, which can interfere with adequate diet therapy, causing metabolic decompensation and contributing to growth retardation.^6^

From 2003 onward, new studies emerged aiming at explaining and improving height deficit in patients with glycogenosis. It was observed that patients with growth retardation had less sensitivity to the growth hormone (GH),^26^ and patients with type Ib presented lower levels of insulin-like growth factor I (IGFI) .^22^ Noto et al.^27^ used GH in a teenager with GSD Ib and achieved an increase in the growth rate. On the other hand, triglyceride and cholesterol levels increased. In addition to GH, other alternatives were analyzed, such as surgical interventions (shunt surgery or liver transplantation), in the correction of growth deficit in patients with GSD I. Although they reduced the deficit in height, no improvement in metabolic parameters was observed.^28^ A study on animals showed the possibility of improvement in growth with gene therapy.^29^In short, several factors may be involved in the etiopathogenesis of growth deficit, including metabolic control, which was not systematically studied in this research.

The present study observed a deficit in muscle mass and, although the patients had an average energy intake above the recommendations and did not present inadequate proteins, no association between anthropometric data was verified, in such a way that other factors may be involved in this process. Some authors suggest that it is possible to improve muscle mass parameters and prevent muscle weakness in patients with glycogenosis by initiating an intensive diet therapy.^19^ ^,^ ^24^

Considering that glycogenosis is a rare disease and difficult to diagnose, the number of patients in the sample was relatively low, which somewhat limits a more precise analysis of the findings. Therefore, the performance of multicenter studies is an alternative to increase the sample. Although data from the statistical analysis were not significant, the results can help understanding the disease.

According to the results, short stature was common in patients with GSD I. Diet therapy failed to reverse the growth deficit in 1/3 of the patients. Moreover, deficit in muscle mass was verified, although there was no reduction in protein intake. Obesity was verified in 1/3 of the patients, possibly justified by the higher energy intake rather than by the excessive intake of carbohydrates and cornstarch, as it would be expected. Considering the scarcity of epidemiological studies on the assessment of the diet of patients with glycogenosis, further research should be carried out in order to improve diet therapy and develop new strategies of adherence to it.
